# Sensorized Vascular High-Fidelity Physical Simulator for Robot-Assisted Surgery Training: A Multisite Pilot Evaluation

**DOI:** 10.3390/jcm15031054

**Published:** 2026-01-28

**Authors:** Giulia Gamberini, Alessandro Dario Mazzotta, Angela Durante, Selene Tognarelli, Niccolò Petrucciani, Gianluca Mennini, Gianfranco Silecchia, Arianna Menciassi

**Affiliations:** 1 Health Science Interdisciplinary Center, Sant’Anna School of Advanced Studies, 56127 Pisa, Italy; 2 The BioRobotics Institute, Sant’Anna School of Advanced Studies, 56127 Pontedera, Italy; 3 The Department of Excellence in Robotics & AI, Scuola Superiore Sant’Anna, 56127 Pisa, Italy; 4 Department of General and Specialist Surgery, Sapienza University of Rome, 00185 Rome, Italy; 5 Fondazione Toscana Gabriele Monasterio, 56124 Pisa, Italy; 6 Department of Medico-Surgical Sciences and Translation Medicine, Faculty of Medicine and Psychology, St Andrea Hospital, Sapienza University of Rome, 00189 Rome, Italy

**Keywords:** high-fidelity physical simulator, vascular structure, discriminant validity, vessel deformation, robotic surgical training

## Abstract

**Background/Objectives**: Robot-Assisted Surgery poses challenges in skill acquisition due to the lack of haptic feedback, which may lead to adverse intraoperative events. This study focused on a multisite pilot evaluation on the simulator’s ability to discriminate between different levels of expertise and the ability to explore potential differences between surgical specialties. **Methods**: We built a simulator that can replicate anatomies of vascular and adipose tissue. A resistive stretching sensor was integrated into a silicone vessel to objectively measure its deformation. A total of 18 males and 12 females, aged between 26 and 64 years old, participated to the study. In total, there were 30 participants, (21 general surgeons, 2 thoracic surgeons, 4 gynecologists, 3 urologists) and they performed two repetitions of a surgical task and filled in a questionnaire about face- and content validities and a system usability scale. The tests were conducted between February and October 2023. **Results**: The discriminant validity was positively assessed, considering the maximum deformation value (*p*-value = 0.0479) and the mean deformation value (*p*-value = 0.0317). Differences were found between urologists, (i) general surgeons (*p*-value = 0.0167) and, (ii) gynecologists (*p*-value = 0.0495). The face- and content validity of the simulator received 80% and 90% of positive answers, respectively. **Conclusions**: Future works will deal with the evaluation of the simulator abilities in surgical training by comparing surgeons trained on the simulator to those who are not.

## 1. Introduction

In recent years, the increasing adoption of Robot-Assisted Surgery (RAS) has resulted in the evolution of training methodologies that improve the role of simulation [1]. Robot-assisted surgical procedures are increasing every year with more than 2,680,000 procedures performed using a da Vinci Surgical System in 2024 (data taken from the Intuitive Surgical Inc., Annual Report). RAS allows us to achieve increased surgical precision, less discomfort after surgery, shorter hospital stays, and faster recovery time, all of which improve patient outcomes and lessen the strain on healthcare resources, which plays a critical role in public health [2]. Due to different control strategies, from more analogic “hands on” systems to digital devices and indirect consoles, the length of the learning curve has increased (40–70 cases to reach proficiency) [3]. Furthermore, the minimally invasive procedures reduced the haptic feedback in robotic surgery [4]. On this matter, a specific new approach is needed to ensure adequate medical training, which will maintain patient safety during robotic surgical procedures [5,6,7]. Since the introduction of simulations in healthcare, the skills required by a robotic surgeon can be trained using different approaches. Simulation training includes dry-laboratory activities, wet-laboratory activities, and virtual-reality (VR) simulators. It offers a risk-free environment for trainees and enables them to practice complex procedures, which can enhance clinical performance and patient outcomes [8,9]. In particular, when dealing with delicate tissues, such as blood vessels, the specific training is much more needed as the application of excessive force may cause intraoperative complications, like massive bleeding or damage to other tissues or organ, which may lead to the immediate conversion to open surgery [10,11,12,13].

The application of a large deformation leads to high strain values, which are a primary cause of vascular rupture and present a considerable risk of intraoperative hemorrhage. In clinical practice, excessive force applied during vessel manipulation, whether sudden or sustained, is a recognized factor contributing to bleeding and may require conversion to open surgery [14,15,16]. In addition, mean deformation is a crucial parameter for assessing the capabilities of handling vessels during the procedure [17,18]. Repeated or prolonged deformation, even without high peak events, can cause microtrauma to the vessel wall. Mechanical stress can trigger inflammation and compromise tissue integrity, increasing the risk of postoperative complications that may cause stenosis or pseudoaneurysm formation and/or anastomotic healing [19,20].

In order to reduce the number of such adverse events, surgeons must be able to visually assess tissue deformation—a concept known as ‘visual haptic’—in addition to precise hand–eye coordination [21]. To acquire visual-haptic competence, structured training is mandatory to achieve the combination of specific knowledge, a safe experience, and a productive reflection in a simulation environment [22,23]

However, few physical simulators are available to ensure the gaining of visual haptics, especially regarding vascular robotic surgery [24]. Thus, the aim of this article is to present a physical simulator for robotic surgery and to perform discriminant validity tests within different pools of end-users.

## 2. Materials and Methods

**Figure 1:**
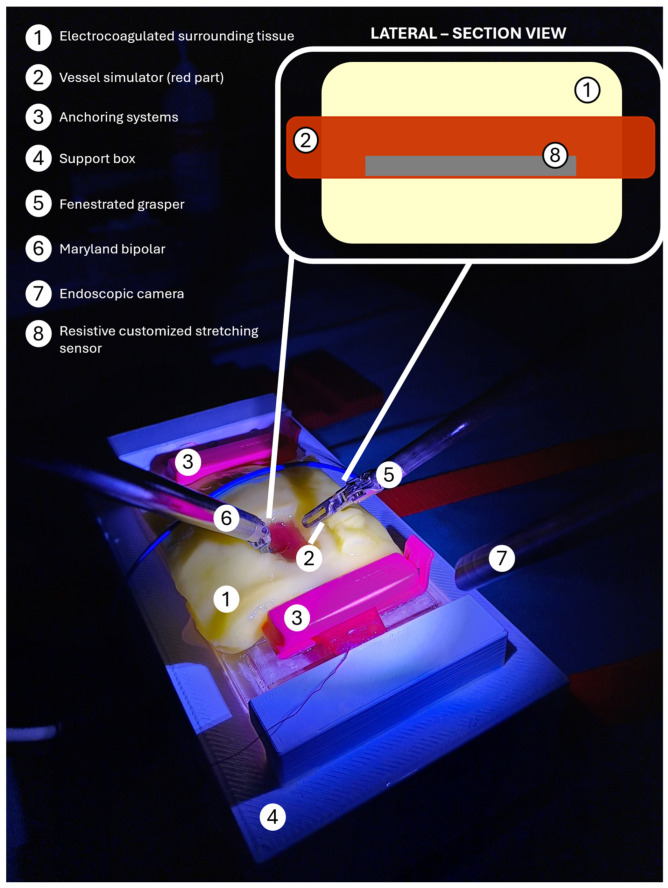
The simulator in the operating room during a testing session with all the system components and the surgical instruments used for the tests.

A high-fidelity physical simulator of the vascular structure was realized as reported in [24]. The simulator prototype under the da Vinci Xi (Intuitive Surgical Inc., Sunnyvale, CA, USA) during the validation tests is shown in Figure 1.

The simulator was meant to teach and train the isolation and resection of vascular structures, which is a common interventional task, while removing a target tissue due to structural abnormalities or tumors. Our simulator was meant to reproduce the structure of a main vein comparable with a pulmonary vein (length of 120 mm, diameter of 13 mm, and wall thickness of 1.5 mm) [25], and it was made through cast molding using silicone Ecoflex 00-30 (Smooth-On, Macungie, PA, USA)—chosen to match the mechanical properties of human tissues, as reported in [19].

In this framework, the specific aims of this study were to assess the following:

(i) the discriminant validity of the simulator—defined in our case as the ability to discriminate between different levels of expertise between novices, fellows, and expert surgeons [26];
(ii) the ability to explore, based on the tests results, the potential differences among the surgical specialties using a stratification of the sample.

### 2.1. Description of the Technical Features of the Vascular Simulator

As stated above, the high-fidelity physical simulator was designed to replicate the anatomic structure of a main vein and its adipose surrounding tissue [24]. The surrounding tissue was realized as a parallelepiped with an elliptical central hole for housing the vessel, and it was made using polyvinyl alcohol (PVA), allowing its electrocoagulation. A support structure was created to securely attach the simulator to the surgical table during the testing sessions and to store all the electronic components.

To measure vessel deformation during surgical manipulation and to evaluate forces induced in the vessel, a customized resistive stretching sensor was integrated into the vein. The sensor is soft, embeddable, and capable of detecting deformation of the vascular structure from 0 to 30% of strain. It is fabricated using a commercial fabric, i.e., Stretch Conductive Fabric (Less EMF, Latham, NY, USA), and deformation signals are acquired, recorded, and stored through a graphical user-interface program realized using LabVIEW 2019 software (National Instruments, Austin, TX, USA). As the training was aimed at assessing only technical manipulation, the blood stream was not included in the simulator.

The vessel and its adipose surrounding tissues were mechanically characterized by means of tensile tests. As shown in [24], our results are similar to 90.4% with respect to the pulmonary porcine vein and were obtained using an Instron machine (Instron, Norwood, MA, USA). The adipose surrounding tissue was characterized by performing compressive tests (controlled by a displacement percentage ranging from 0% to 90% at a rate of 200 mm/min) and it showed a Young’s modulus of 27.34 ± 2.79 kPa, that is comparable with the human adipose organ-surrounding tissue [27].

### 2.2. Population

A pre-statistical analysis was performed to determine the desired sample size. G*Power 3.1.9.7 software [28] was used and an *ANOVA: Fixed-effects, omnibus, one-way* was conducted with the following parameters:

- Effect size equal to 0.6,
- α = 0.05 (representing the significance level),
- Power equals 0.8,
- Number of groups equal to three.

The sample size was computed a priori, and we determined that the total sample size should be equal to 30 people.

Participants were selected from two academic institutions: the Research Institute Against Digestive Cancer (IRCAD), Strasbourg, France, and the Azienda Ospedaliera Universitaria (AOU) Sant’Andrea, La Sapienza, Rome, Italy. Specific ethical committee approvals were obtained, i.e., IRCAD—New Devices 2022–2025 and Sant’Anna School of Advanced Studies Authorization n°37/2023. All the experiments were performed in accordance with the General Data Protection Regulation (GDPR). All the recruited surgeons signed an informed consent form before starting the experiments. The evaluation was conducted in an operating theater using a da Vinci Xi surgical robot.

The surgeons were divided into three different groups according to the length of experience declared as the primary operator in RAS. They were classified as follows:

- Novices: Junior residents with no RAS experience (0 years of RAS experience),
- Fellows: Senior residents and young surgeons with medium experience (1–2 years of experience in RAS),
- Experts: Surgeons with extensive RAS experience (at least 3 years of RAS experience).

In addition, participants were selected from different surgical specialties using a maximum variation sampling between those available in the hosting hospitals (general surgery, thoracic surgery, gynecological surgery, and urological surgery) [21]. The general surgery population includes surgery of the digestive system, oncological surgery, emergency surgery, endocrine surgery, reconstructive surgery, and organ transplant surgery.

### 2.3. Protocol

At the beginning of the testing session, all participants received a brief description of the surgical task through a slide presentation and a standardized instructional video; then, the study objectives and the safety- and the data-protection information were presented. After these preliminary phases, participants who were willing to participate in the tests were asked to sign an informed consent form. Demographic information and years of experience in RAS were collected anonymously using a questionnaire.

Participants underwent a familiarization session lasting 5 min. The familiarization session relied on guided steps to allow the users to understand the task workflow, the equipment, and the requirements. Moreover, they were instructed to operate the da Vinci Surgical System in safe conditions.

The simulated surgical task comprised three main steps:

1. Dissection and isolation of a target vessel.
2. Passage of vessel loops under the vessel to pull it up.
3. Insertion of a robotic stapler for vessel transection.

Two separate repetitions of the entire task per participant were performed. Sensor data were recorded to evaluate participants’ performance metrics that included the maximum vessel deformation, reached during the simulated surgical task, and the mean vessel deformation along the surgical task. Both maximum- and mean deformation signals were used as performance indicators of clinical competence during the simulated surgical task. At the end of the surgical tasks, the surgeons were asked to fill in an anonymous ad hoc questionnaire. The questionnaire contains 22 items, divided into three main sections. The three sections were about the face validity of the simulator, the content validity of the simulator, and the system usability scale (SUS). The face- and content validities were evaluated using Messick’s contemporary framework [29]. The other aspects of Messick’s framework were not considered in this work, as they do not apply to our specific case. Face validity, also known as Evidence based on Response Process Validity [30], is the subjective view end-users have of how realistic a simulator is [31]. Content validity, or Evidence based on Test Content validity [32], is defined as the extent to which a simulator’s content is representative of the knowledge or skills that have to be learned in a real clinical setting [33]. The SUS is a reliable, zero-cost psychometric tool composed of 10 alternating positive- and negative-formulated statements for which a respondent gives a subjective evaluation of a system’s usability [34]. The complete list of items divided into three sections is reported in Table 1. The answers were recorded through a Linkert scale ranging from 0 to 5, where 0 represents very-unrealistic-for-the-face-validity items and strongly-disagrees-with-the-content-validity-and-SUS items, while 5 represents very-realistic-for-the-face-validity items and strongly-agrees-with-the-content-validity-and-SUS items.

### 2.4. Statistical Analysis

#### 2.4.1. Sensor Data Statistical Analysis

To evaluate discriminant validity and assess the ability of the simulator to distinguish between different surgical specialties, the Kruskal–Wallis ANOVA test [28] was performed in MATLAB R2024b software (MathWorks, Natick, MA, USA). Whenever statistically significant results were achieved, post hoc analyses were performed.

#### 2.4.2. Questionnaire Data Statistical Analysis

In addition, a descriptive statistical analysis was performed for the validity items of the face and content, as well as for the SUS questions. To evaluate the overall internal consistency of the questionnaire for each of the three sections, McDonald’s omega coefficient was used [35]. For the content validity items, additional analyses were performed:

- Calculation of the *Content Validity Index* (*I-CVI*) defined as the proportion of surgeons who rated an item as relevant.
  $$I\text{-}CVI=\frac{\text{Number of surgeons rating}\;4\;\text{or}\;5}{\text{Total number of surgeons}}$$

- Calculation of the *Scale-level CVI* (*S-CVI*) defined as the average of the *I-CVIs* across items [32].

Regarding the SUS, the total SUS score was calculated. The SUS is usually made up of both positive- and negative constructed elements; therefore, the total SUS score was calculated according to the literature [30] by reversing the negative elements, summing all the scores obtained, and scaling it to 100. In particular, the following should be considered:

- Negative items are considered as 5−values achieved.
- Positive items are considered as values achieved−1.

## 3. Results

In total, 30 surgeons participated (13 novices, 8 fellows, and 9 experts). The main features of the three groups in terms of age, sex, dominant hand, specialties, and clinical-training experience in the simulation are reported in Table 2.

Although we tried to obtain at least one representative level of experience and/or surgical specialties (general surgery, thoracic surgery, gynecology, and urology), in the expert group, thoracic surgeons and urologists were missing. This was mainly due to the inaccessibility of experienced surgeons in these specialties in the surgical groups contacted. Regarding simulation experience, in the novice group, 69% of the subjects had previous experience with simulators for clinical training. In the fellow- and in the expert groups, this percentage reaches 88% and 100%, respectively.

**Figure 2:**
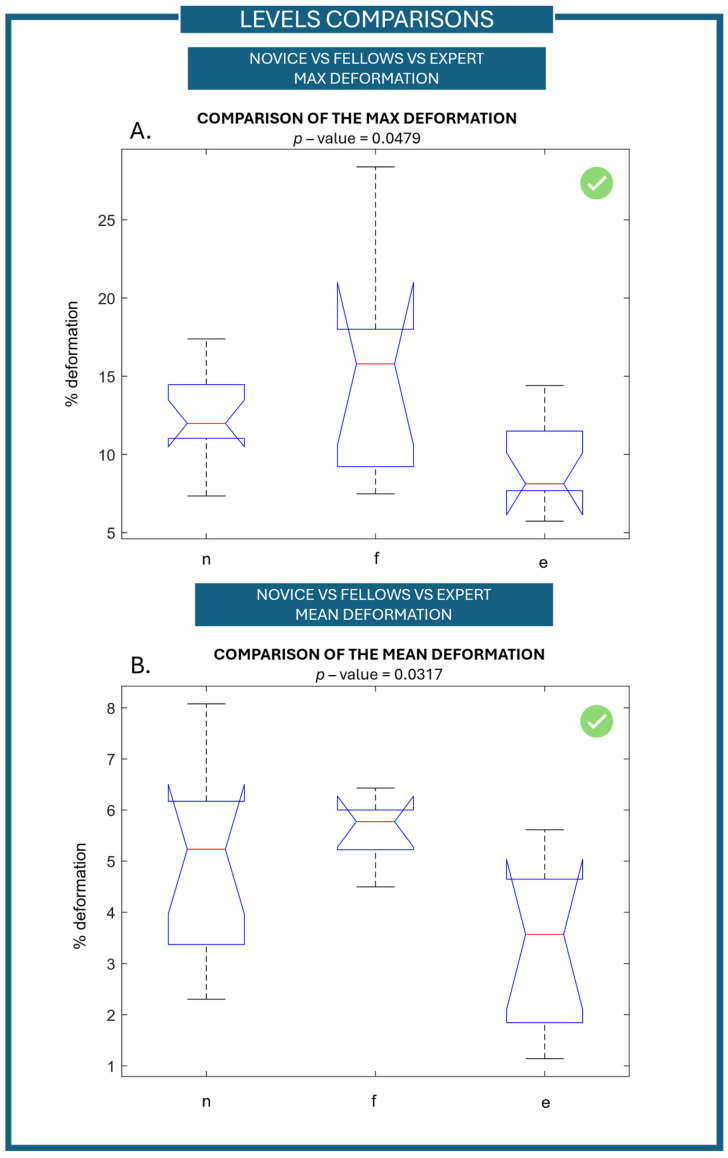
Comparison of the outcome measures between the three groups of experience (n: novice, f: fellow, e: expert). (**A**) Comparison of the maximum value of the deformation signal during the first repetition between the three groups of experience. (**B**) Comparison of the mean value of the deformation signal during the second repetition between the three groups of experience.

**Figure A1:**
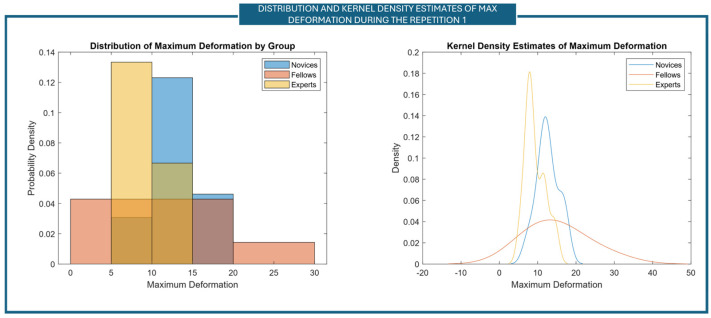
Data distribution of maximum deformation divided per novices (light blue), fellows (light pink) and experts (light yellow) is depicted on the left, while on the right, the kernel density estimates are plotted. According to the analysis, data did not differ significantly from a normal distribution.

**Figure A2:**
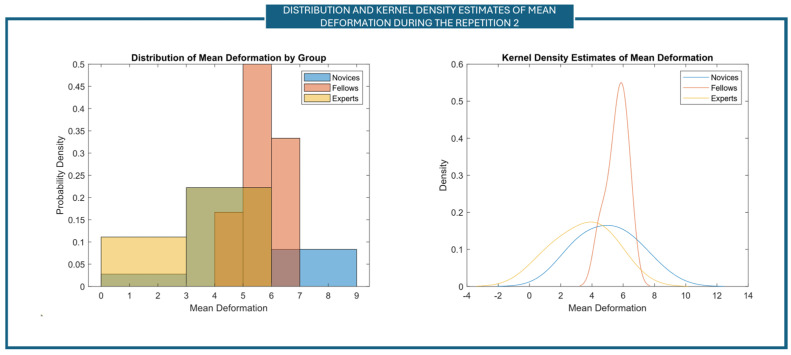
Data distribution of mean deformation divided per novices (light blue), fellows (light pink) and experts (light yellow) is depicted on the left, while on the right, the kernel density estimates are plotted. According to the analysis, data did not differ significantly from a normal distribution.

Based on the sensor data, the vessel simulator was able to distinguish between the three different levels of experience, i.e., novices, fellows, and experts. Considering the maximum value of vessel deformation derived from continuously recorded data during the first repetition of the task, a statistically significant difference between the three groups was found—*p*-value equal to 0.0479, as shown in Figure 2A. The distribution of the data was evaluated, confirming a distribution not significantly different from a normal distribution (18% of difference). Thus, to verify whether the use of a non-parametric test was appropriate, Levene’s test for equality of variances was performed. A *p*-value of 0.018 was found, indicating that variances differ; therefore, the use of a non-parametric test is consistent with our aim (the full analysis of data distribution and variance is reported in Appendix A, Figure A1). The post hoc statistical analysis did not highlight any significant differences overall; however, when considering the individual pairs (experts vs. novices, experts vs. fellows, and fellows vs. novices), a statistically significant difference was found between experts and novices, with a *p*-value of 0.0178. Moreover, a statistical significance difference between the three levels of experience was also found by considering the mean value of the vessel deformation during the second task-repetition with a *p*-value of 0.0317 (Figure 2B). Data distribution does not differ significantly from a normal distribution (12% of difference). A *p*-value of 0.07 was found performing Levene’s test for equal variances; thus, variances do not differ, and neither a non-parametric test nor ANOVA can be used. To evaluate all the metrics with the same methodology, a non-parametric statical test was kept as evaluation performance metrics (the full analysis of data distribution and variance is reported in Appendix A, Figure A2). From the post hoc analysis, a statistically significant difference was found between mean ranks of expert and fellows (*p*-value = 0.0067).

By considering the results presented here, the discriminant validity in terms of levels of expertise was positively assessed. In addition, using the recruitment modality, the collected data was analyzed moving from surgical expertise to surgical specialties. An additional comparison was carried out by dividing the simulator data collected during the tests into four different groups, one for each clinical specialty only. This additional analysis was carried out to determine whether the simulator was also able to distinguish between them. A statistically significant difference cannot be achieved (*p*-value of 0.2057) when comparing all the specialties together. This could be justified by the unequal distribution of the users in the four specialties groups.

**Figure 3:**
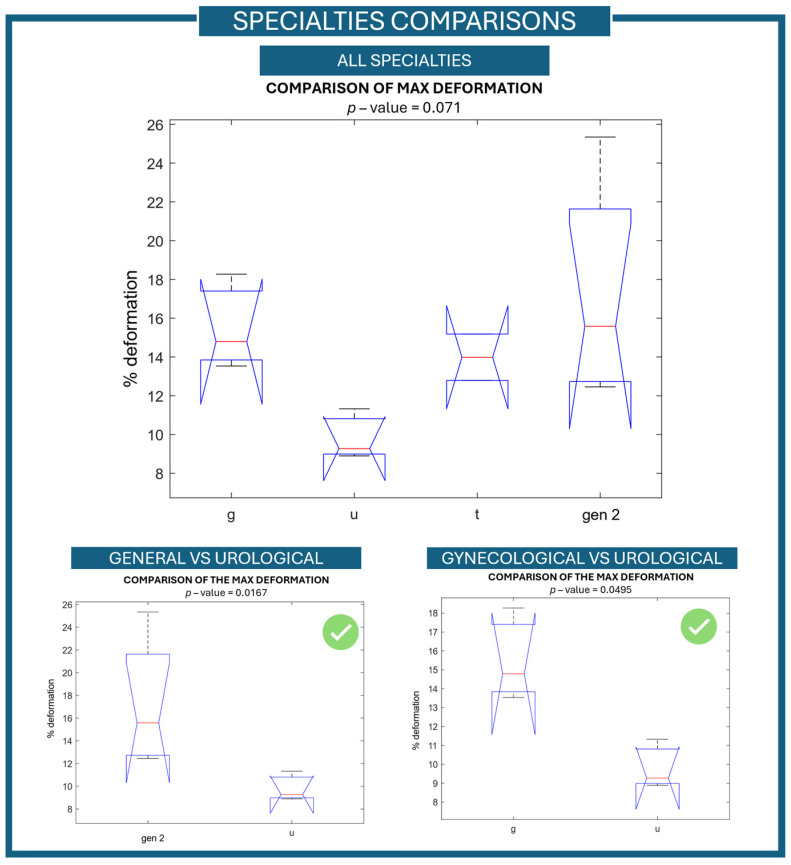
Specialties comparison of the outcome measure. The comparison between all four types of specialties (gen 2: general surgery 2, u: urology, g: gynecology, t: thoracic surgery) is reported on the top part of the figure together with its *p*-value. In the bottom part of the figure, the couple’s testing results having a statistically significant difference are reported.

Due to the large difference in users’ numbers, and to better understand the ability of the simulator to discriminate between the different surgical specialties, a reduced sample of general surgeons was considered. By considering the number and the expertise distribution (percentage data are reported within round bracket in Table 2) of the other surgical specialties, a proportional number of general surgeons was randomly selected. By using a random-generator number extraction, four novices, two fellows, and one expert surgeon were randomly selected from the general-surgery population by performing a random under sampling in accordance with the literature [36]. It is important to note that the differentiation of the levels of the intra-user group was maintained. Then, this new user group (general surgery 2) was used to evaluate the ability of the simulator to differentiate between different surgical specialties. Nevertheless, no statistically significant difference was achieved (*p*-value = 0.071), Figure 3—top part. The failure to reject the null hypothesis of samples coming from different populations’ distribution could again be linked to the limited size of the population groups. A higher number of surgeons should be involved to further analyze the capabilities of the simulator in differentiating between surgical specialties.

However, to further analyze the ability of the simulator to discriminate between surgical specialties, a couples’ analysis was performed. In particular, the six different combinations of pairs of surgical specialties were compared and analyzed. Maximum vessel deformation data were used as reference during the second repetition of the surgical task (after familiarization tasks). This choice was made to reduce the effects of performance bias and variability due to unfamiliarity that may impact the initial attempt, typically affected by the user’s adaptation to the simulator and the surgical procedure.

The pairs tested are

- General surgery vs. thoracic surgery;
- General surgery vs. gynecological surgery;
- Gynecological surgery vs. thoracic surgery;
- Gynecological surgery vs. urological surgery;
- General surgery vs. urological surgery;
- Urological surgery vs. thoracic surgery.

Based on the analysis performed, a statistically significant difference was found between gynecological and urological surgeries (*p*-value of 0.0495) and between general and urological surgeries (*p*-value of 0.0167).

**Figure 4:**
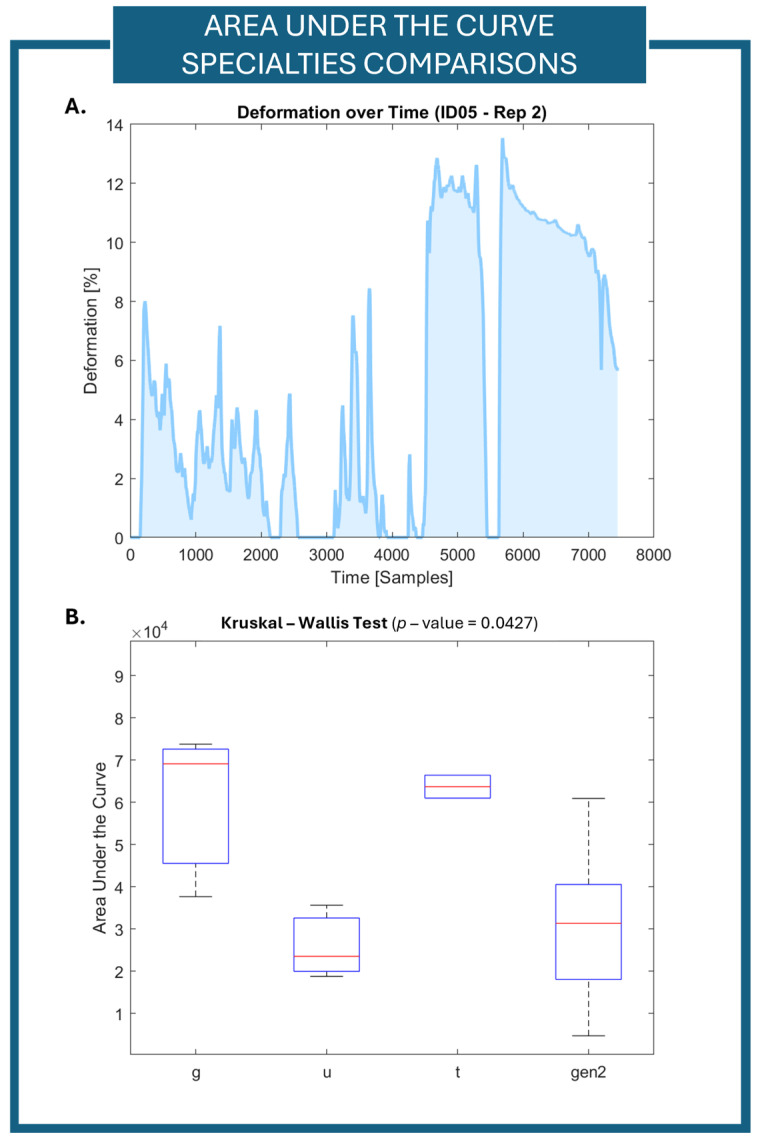
(**A**) Example of deformation curve. The light-blue line is the deformation over time, while the filled space below is the area under the curve considered. (**B**) Boxplot showing the area under the curve distribution for each surgical specialty.

The results of the analysis for the comparisons of surgical specialties are depicted in Figure 3. For the analysis of the couple, only those with a statically significant difference were reported. In addition to maximum- and mean metrics, the area under the deformation–time curve (Figure 4A) was also analyzed in order to capture the cumulative strain applied to the vessel over the duration of the task.

From the analysis of the area under the curves, a statistically significant difference when comparing the different surgical specialties during the second repetition of the task was found (*p* = 0.0427), as reported in Figure 4B.

**Figure 5:**
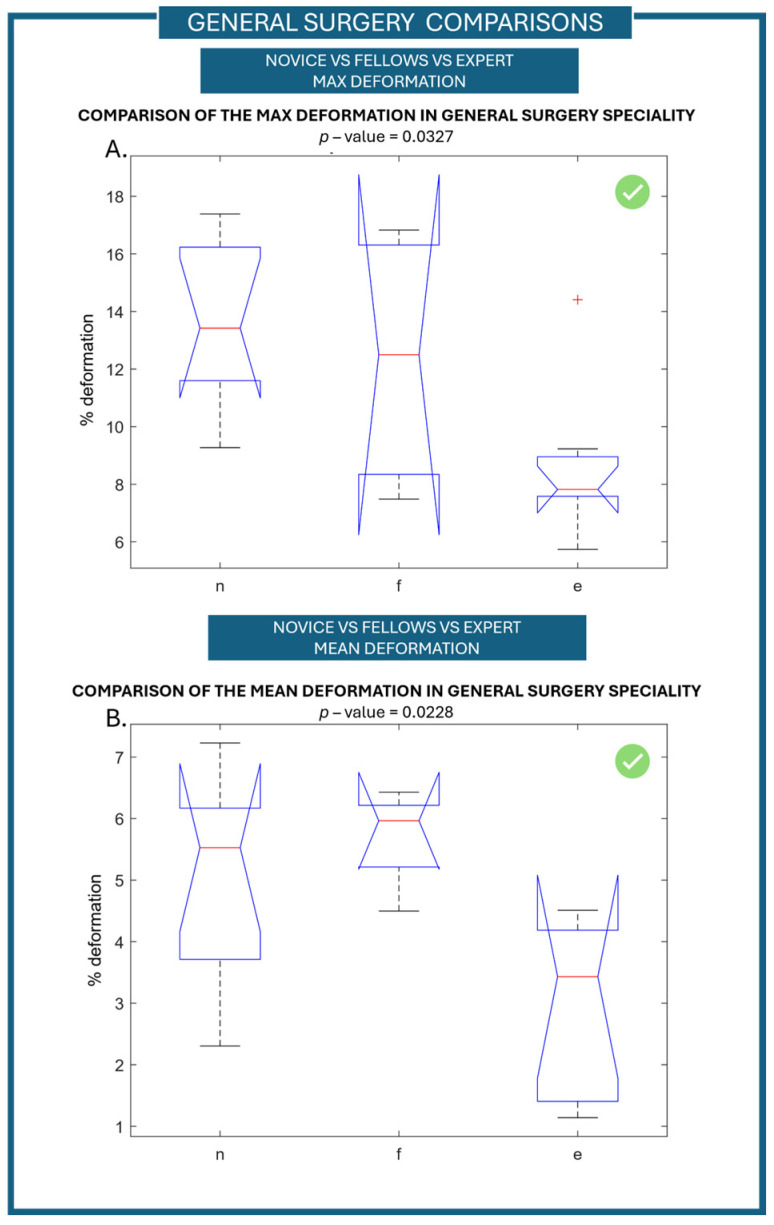
Comparison between the different levels of expertise in general surgery (n: novice, f: fellow, e: expert). (**A**) Comparison of the maximum value of the deformation signal between the three groups of experience during the first repetition. (**B**) Comparison of the mean value of the deformation signal between the three groups of experience during the second repetition.

Finally, as mentioned above, general surgery was the specialty most represented in our population samples (21 users). To further assess the ability of the simulator, a comparison analysis restricted to only the general-surgery specialty was carried out. In particular, a comparison between the different levels of expertise in the general-surgery population was made through the Kruskal–Wallis ANOVA test. From the results obtained, a statistically significant difference between novices, fellows, and experts was found when considering the maximum deformation value of the vascular structure during the first repetition—*p*-value of 0.0327 (Figure 5A). Moreover, the difference in the mean vessel deformation during the second repetition was found to be statistically significant between the three levels of expertise with a *p*-value of 0.0288 (Figure 5B). From the post hoc analysis, a statistically significant difference was found between experts and novices in the maximum value of the first repetition, while for the mean value of the second repetition, a statistically significant difference was found between experts and fellows.

This finding can be explained by a procedural reason. In the initial repetition, higher vessel-deformation values—especially in the novice and fellow groups—can be attributed to their unfamiliarity with the surgical task. At this stage, less-experienced users often apply excessive or inadequately moderate force, leading to an increased maximum-deformation of the vascular structure. This behavior aligns with preliminary actions and an absence of advanced haptic perception and motor coordination.

In the second repetition, a performance-stabilization effect is noted: participants, having been previously exposed to the task, begin to modify their interaction with the simulator. As a result, peak deformation values become more consistent and no longer differ significantly among experience levels, while the mean deformation—reflecting overall precision and control—emerges as the more-sensitive parameter for discriminating between levels of expertise. This shift supports the simulator’s responsiveness not only to initial technical gaps but also to short-term learning- and adaptation processes.

**Figure 6:**
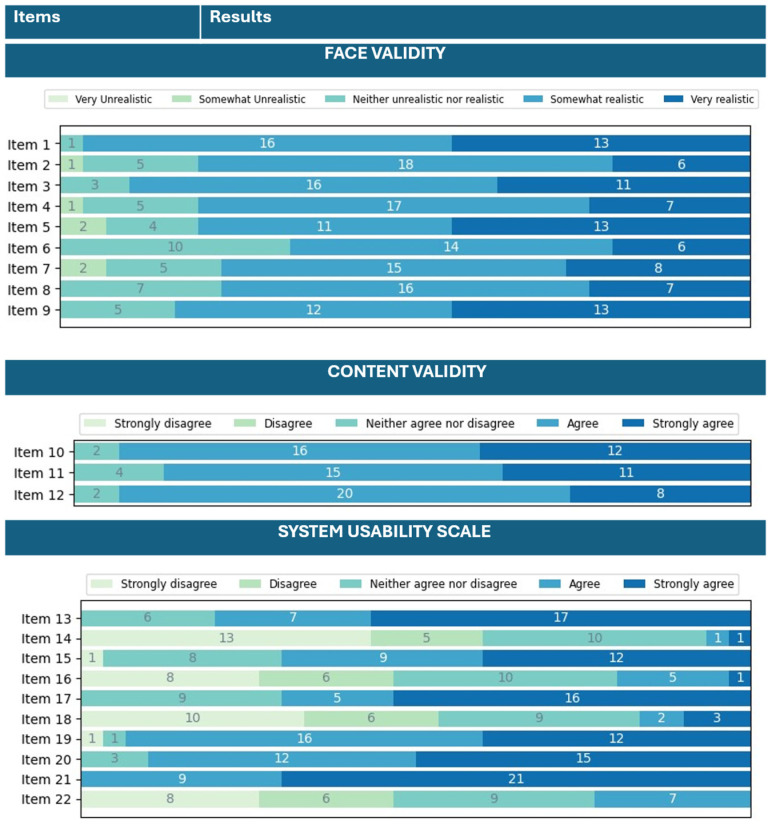
Questionnaire results regarding the face- and content validities and the system usability scale. The questions left unanswered were counted as “neither unrealistic nor realistic” or “neither agree nor disagree”.

The analysis of the results of the questionnaire—administered after the testing session—about evidence based on response processes (face validity), evidence based on test content validity, and the system usability scale is reported in Figure 6. Considering the face validity of the simulator, the overall global impression of the simulator was positively evaluated, with 29 surgeons declaring the simulator to be somewhat realistic or very realistic. Regarding the realism of the anatomical components, the simulator with the adipose tissue was considered more realistic than the version without the adipose tissue (Item 3 was rated higher than Item 2). Both the visual appearance of the vein and the adipose tissue (Items 4 and 5) were positively evaluated (somewhat realistic or very realistic) by 24 surgeons (80%). The haptic feedback when manipulated with hands, of both the vein and the adipose tissue, was evaluated positively by 20 and 23 surgeons (Items 6 and 7), while the interaction between the surgical instruments was positively evaluated by 23 and 25 surgeons for the vein and the adipose tissue, respectively (Items 8 and 9).

Regarding the content validity (Items 10–12), the simulator was thought to be useful in teaching:

- Vascular structure isolation by 28 surgeons;
- The stapling of vascular structure by 26 surgeons;
- To minimize the forces applied to vascular structures by 28 surgeons.

The system usability scale of the simulator was performed to evaluate the ease of use of the simulator and the perceived availability in using the simulator by end-users. Twenty-four surgeons declared that they would like to use the simulator as a training platform (Item 13). Only two surgeons found the simulator more complex than expected (Item 14). Twenty-one surgeons thought that the simulator was easy to use (Item 15). Only six declared they would need a technician to support them in using the simulator, and five found difficulties in understanding the electronics connections (Items 16 and 18). The graphical user-interface was found understandable and clear by 21 surgeons (Item 17). A total of 28 and 27 users think that most of their colleagues will learn how to use the simulator very fast and that the system is intuitive and “plug and play” (Items 19 and 20). All surgeons agreed on being comfortable with the use of the simulator. Only seven surgeons declared that they would need more time to become familiar with the setup of the simulator.

Descriptive statistical analyses and McDonald’s omega analyses were performed both for face- and content validities, as well as for the system usability scale. Evidence based on response processes validity was used to evaluate whether the respondents interpreted the realism of the anatomical structures, mechanical haptics, and instrument–tissue interactions.

**Figure A3:**
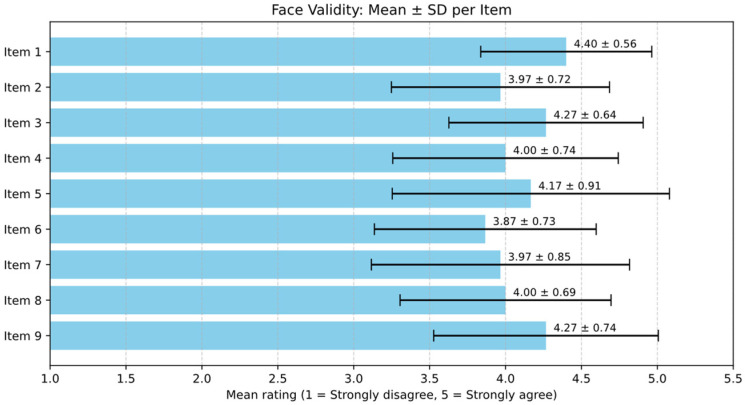
Histogram plot of the mean and standard deviation of the results achieved for the face validity (Item 1 to Item 9).

Table 3 reports the values of the mean and standard deviation for each item from one to nine (which are related to the evidence based on response processes validity). This is also graphically depicted in Figure A3. By the calculation of the McDonald’s omega for evidence based on response processes validity, we achieved an omega value equal to 0.978, thus representing an excellent internal consistency of the questionnaire items [37,38,39].

**Figure A4:**
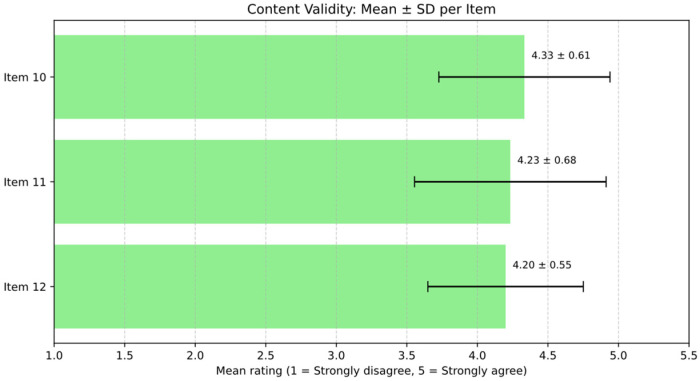
Histogram plot of the mean and standard deviation of the results achieved for the content validity (Item 10 to Item 12).

In Table 4, the values of the mean and standard deviation for each item from 10 to 12 (which are related to the evidence based on test content validity) are reported. This is also graphically depicted in Figure A4. By the calculation of the McDonald’s omega, for evidence based on response processes validity, we achieved an omega value equal to 0.940, thus representing an excellent internal consistency of the questionnaire items [37,38,39].

In addition, for the content validity, content validity indexes were calculated for each item of the questionnaire:

- I-CVI for item 10: 0.933.
- I-CVI for item 11: 0.867.
- I-CVI for item 12: 0.933.

All the items have a CVI index greater than 0.78; thus, the items have excellent content validity, and they can be retained. Regarding the scale-level CVI that considers the average of all the I-CVI items, we achieved a S-CVI equal to 0.911 [32].

**Figure A5:**
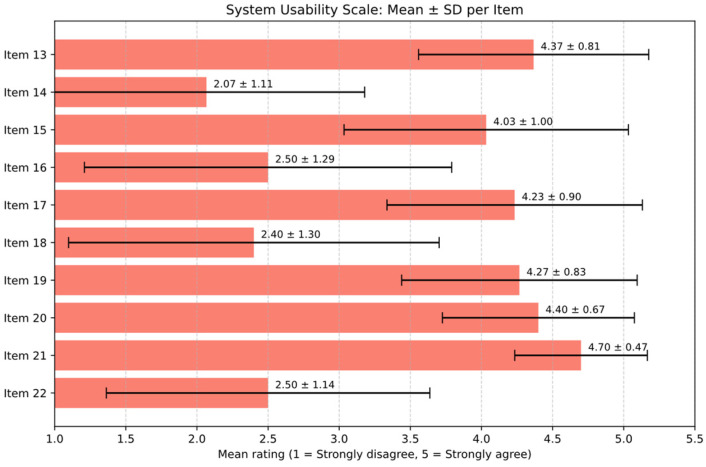
Histogram plot of the mean and standard deviation of the results achieved for the System Usability Scale (Item 13 to Item 22).

Table 5 reports the values of the mean and standard deviation for each time from 13 to 22 (which deal with the usability of the simulator). This is also graphically reported in Figure A5. By the calculation of the McDonald’s omega, for SUS we achieved an omega value equal to 0.978, thus representing an excellent internal consistency of the questionnaire items [32,33,34]. Moreover, the total SUS score was calculated, achieving a score equal to 75.92, showing an average usability of the system [40].

## 4. Discussion

This paper presents the pre-clinical assessment of a soft sensorized high-fidelity physical vascular structure with simulated adipose tissue by involving 30 surgeons with different levels of experience in RAS working in different surgical specialties. The simulator was designed to enable an objective- and proctor-independent evaluation of robotic-assisted surgical manipulation of delicate tissues, thanks to a resistive, customized stretching sensor embedded into the silicone vascular structure able to detect the vessel deformation through the simulated surgical task.

Although distinguishing between experience classes—particularly among experts, fellows, and novice—presents challenges and it is not usually presented, a statistically significant difference in maximum tissue-deformation was identified across the three users’ groups. Moreover, a statistically significant difference in mean deformation data was found for experts, fellows, and novices. This emphasizes the potential of sensor-based simulators to offer quantifiable metrics that can be used to objectively distinguish surgical-skill levels, thereby enabling targeted enhancements to training programs.

One of the main challenges of this study was recruiting fellows and expert surgeons, who are often difficult to involve. Fellows are in a transitional phase between training and independent practice, so their availability is limited. Expert surgeons are usually busy with clinical duties, which also makes their participation harder. As a result, these groups are often underrepresented, making it difficult to balance the sample size across different experience levels [41,42].

One of the main goals achieved by this work was the ability to extend the investigation to all three categories of surgeons, revealing significant differences in performance when comparing their data to those of both novices and experts. Future research could benefit from expanding recruiting criteria or collaborating with additional training facilities to obtain a more representative sample of fellows, thereby strengthening the validity of data comparisons across various experience levels. On the other hand, involving additional training facilities could be a solution for increasing the number of expert surgeons involved in the analysis.

In addition to the analysis based on surgical expertise, the study examined the simulator’s ability to differentiate among different surgical specialties [43]. Two pairwise comparisons demonstrated statistically significant differences in maximum vessel-elongation during the second task repetition: general surgery (gen2, n = 7) versus urology (n = 3) (*p* = 0.0167) and gynecology (n = 4) versus urology (n = 3) (*p* = 0.0495). This difference may be ascribed to the differing anatomical contexts and tissue types often encountered in their respective practices.

General surgeons frequently operate on tissues like the mesentery or bowel that exhibit relative mobility and are more amenable to manipulation. In contrast, urologists typically perform procedures in anatomically confined and fibrotic spaces, such as the retroperitoneum and pelvis, which demand a high degree of precision and delicate tissue handling. A similar pattern was noted between gynecologists and urologists, where the difference also reached statistical significance. Although both professions often function within the pelvis, the anatomical structures they engage with vary significantly. Gynecologic procedures usually involve more compliant responsive tissues, such as the uterus and adnexa, whereas urologic interventions involve denser- and less-elastic structures like the bladder and prostate. Nonetheless, these results should be regarded with caution because of the limited sample sizes of the urologic- and gynecologic cohorts. The statistical significance indicates the simulator’s ability to differentiate modest inter-specialty changes in tissue-manipulation behavior; nevertheless, more research with larger, more balanced populations is necessary to validate these findings and investigate their therapeutic implications more thoroughly [44,45].

Additionally, the comparison of the discriminant validity of the simulator when restricted to general surgery was carried out, and a statistically significant difference was found.

The obtained results are in line with the literature indications; Cundy P. et al. highlighted that (i) simulators can be used to enhance skills related to force sensing, and (ii) surgical skills are influenced by expertise level [45,46,47]. Our results confirm that surgical skills are influenced by the levels of experience. In fact, a statically significant difference was found between the three expertise levels. Future works will deal with the evaluation of the simulator abilities to enhance skills related to force sensing by comparing surgeons trained on the simulator to those who are not. In addition, thanks to the high-fidelity features of the simulated scenario, the proposed simulator allowed surgeons to train and test on a model that, not only from a mechanical point of view but also from a visual appeal, replicates the properties of human tissues, and thus allows them to train the so-called visual haptic. Moreover, even with the small number of involved users, statistically significant results were obtained. However, a larger number of surgeons could better demonstrate the training process highlighting more evident skill progression and reducing variability in statistical power. Moreover, a more balanced sample size across the different experience levels and the different surgical specialties could affect the statistical power of the analysis.

In addition, the increased adoption of artificial intelligence (AI) is also expanding in the field of surgical simulation [48]. AI-driven systems can include virtual-reality and augmented-reality surgical training modules as well as video-based assessment tools that can evaluate technique and efficiency, helping trainees to practice skills repeatedly in safe and controlled environments [49,50]. In our specific case, since the simulator is equipped with both visual and acoustic feedback to alert the user when a predefined strain-threshold is exceeded, it would be interesting to integrate artificial intelligence into training courses for clinicians by developing an AI-driven algorithm that adapts the deformation safety-threshold to the surgeon’s performance metrics.

From the questionnaire analysis, the overall global impression of the simulator was positively evaluated by 29 surgeons out of 30. The simulator with the adipose surrounding tissue was appreciated for the realism and the possibility of electrocoagulation given by the PVA-based tissue. The simulator’s face validity, or process-based validity evidence, was positively evaluated, with an average of 80% positive responses. With an average of 27 surgeons out of 30, the simulator was thought to be useful in teaching vascular-structure isolation, the stapling of the vessel, and the minimization of the forces applied. By considering these results, the content validity of the simulator was positively assessed. From the results of the SUS questions, the system was positively evaluated in terms of usability and ease of use. A total of 80% of the surgeons declared that they would like to use the simulator as a training platform, and 70% declared that the graphical user-interface was easy to understand. Overall, the total SUS score was equal to 75.92. In addition, all the surgeons were comfortable in using the simulator; thus, by considering the results reported above, the system usability was positively assessed.

## 5. Conclusions

To conclude, the work presented a multisite pilot evaluation of a sensorized high-fidelity physical simulator of vascular structure. The simulator was tested by 30 surgeons who have different levels of expertise and work in a variety of surgical fields.

A statistically significant difference in maximum- and mean tissue-deformation data was identified for experts, fellows, and novices. Moreover, statistically significant differences in maximum vessel-elongation during the second task repetition between general surgeons and urologists and between gynecologist and urologist were found.

In addition, from the questionnaire analysis, the overall global impression was positively evaluated by 29 surgeons out of 30. The simulator’s face validity obtained an average 80% of positive responses and the content validity was positively assessed. In addition, all the surgeons were comfortable in using the simulator; thus, the system usability was positively assessed.

The results align with the literature, confirming that surgical skills significantly vary with experience level. The high-fidelity simulator effectively supports training by realistically replicating mechanical- and visual tissue properties, enabling the development of force sensing and visual-haptic skills. Although statistically significant results were achieved with a small sample, larger and more balanced cohorts are needed to better demonstrate skill progression and improve statistical power.

Additionally, while the maximum- and mean deformation data provided quantifiable measures of tissue manipulation skills, they represent just some aspects of surgical proficiency. Other aspects, such as decision-making, also under pressure, and error correction were not assessed in this study. Including a broader range of evaluation criteria, a more comprehensive understanding of the competencies developed through robotic-assisted surgery training will be reached. Finally, from a technical point of view, additional efforts will be dedicated to fully replicating the complexity of a live surgical environment. Factors such as real-time bleeding, patient-specific anatomical variations, and more complex vascular structures have to be added to the current system to improve the training reality and potential translation to clinical settings.

Lastly, tracking the surgeons’ movements to understand the correlations between his/her arms movement and the vessel deformation could be an interesting future perspective of the proposed study. Motion-tracking sensors are becoming increasingly popular in medicine as a method for evaluating surgical psychomotor abilities. These sensors have been employed in numerous studies to compare the performance of novices and experts [51,52]. Combining motion tracking with strain measurement could further enhance the simulator’s ability to capture differences in skill levels, offering a more comprehensive understanding of a surgeon’s technical capabilities. This would also offer the possibility of translating clinical-simulation experience to the surgical environment.

## Figures and Tables

**Table 1:** Post-testing questionnaire.

Items	Question or Statement
FACE VALIDITY	
Item 1	Overall global impression of the simulator
Item 2	Anatomical realism without cover with the adipose/connective tissue
Item 3	Anatomical realism with cover with the adipose/connective tissue
Item 4	Visual appearance of the vein
Item 5	Visual appearance of the connective/adipose tissue
Item 6	Haptic feedback of the vein
Item 7	Haptic feedback of the connective/adipose tissue
Item 8	Realism in instrument—vein interaction
Item 9	Realism in instrument—tissue interaction
CONTENT VALIDITY	
Item 10	The simulator is useful in teaching vascular-structure isolation
Item 11	The simulator is useful in teaching the stapling of vascular structure
Item 12	The simulator is useful in teaching to minimize the forces applied to vascular structures
SYSTEM USABILITY SCALE	
Item 13	I think I would like to use this platform for training
Item 14	I found the functioning of the simulator more complex than what I was thinking
Item 15	I think the simulator is easy to use
Item 16	I think I will need a technical person to support me to be able to use the simulator
Item 17	I found the interface functionalities understandable and clear
Item 18	I found difficulties in understanding the physical links to set up and use the simulator
Item 19	I imagine that most of my colleagues will learn how to use the simulator very fast
Item 20	I found the system intuitive and “plug and play”
Item 21	I was comfortable with the use of the simulator
Item 22	I think more time is needed to get familiar with the setup of the simulator

**Table 2:** Characteristics of the three groups. () = %, [] = age range, F = Female, M = Male, VR = Virtual Reality.

	Novice (n = 13)	Fellow (n = 8)	Expert (n = 9)
Age (years)	[29–51]	[26–55]	[26–64]
Sex M	9	7	2
Sex F	4	1	7
Right dominant hand	13	7	9
Specialty	13 (100)	8 (100)	9 (100)
General surgery	9 (69)	5 (63)	7 (77)
Thoracic surgery	1 (50)	1 (50)	0 (0)
Gynecology	1 (25)	1 (25)	2 (50)
Urology	2 (66.6)	1 (33.4)	0 (0)
Simulation training	9 (69)	7 (88)	9 (100)
VR simulation	3 (33.3)	3 (43)	2 (22)
Physical simulation	3 (33.3)	1 (14)	1 (11)
Both	3 (33.3)	3 (43)	6 (67)

**Table 3:** Mean and standard deviation for each item from 1 to 9 of the face validity or evidence based on response processes validity.

	Item 1	Item 2	Item 3	Item 4	Item 5	Item 6	Item 7	Item 8	Item 9
**Mean**	4.41	3.97	4.28	3.97	4.16	3.94	4.06	4.10	4.35
**SD**	0.56	0.69	0.63	0.74	0.92	0.73	0.85	0.66	0.71

**Table 4:** Mean and standard deviation for each item from 10 to 12 of the content validity.

	Item 10	Item 11	Item 12
**Mean**	4.33	4.23	4.20
**SD**	0.61	0.68	0.55

**Table 5:** Mean and standard deviation for each item from 13 to 22 of the system usability scale.

	Item 13	Item 14	Item 15	Item 16	Item 17	Item 18	Item 19	Item 20	Item 21	Item 22
**Mean**	4.37	2.07	4.03	2.50	4.23	2.40	4.27	4.40	4.70	2.50
**SD**	0.81	1.11	1.00	1.29	0.90	1.30	0.83	0.67	0.47	1.14

## Data Availability

The raw data supporting the conclusions of this article will be made available by the authors on request.

## Appendix A

Data distribution divided into groups of the experience’s maximum deformation achieved during the first repetition is depicted in Figure A1 left, while the Kernel Density estimates are reported in Figure A1 right.

The data distribution, divided into groups based on the mean deformation achieved during the second repetition, is shown in Figure A2 (left), while the Kernel Density estimate is reported in Figure A2 (right).

The histogram plots of the different ratings of the questionnaire items are reported below. Figure A3, Figure A4 and Figure A5 illustrate the evaluation results: Figure A3 presents face validity, Figure A4 presents content validity, and Figure A5 presents the System Usability Scale scores.
